# Complete Genome Sequence of a Human Enterovirus 71 Strain Isolated in Brunei in 2006

**DOI:** 10.1128/genomeA.00522-13

**Published:** 2013-07-18

**Authors:** Zainun Zaini, Peter McMinn

**Affiliations:** Virology Unit, Department of Laboratory Services, Ministry of Health, Bandar Seri Begawan, Bruneia; Infectious Diseases and Immunology, The University of Sydney, Sydney, Australiab

## Abstract

The complete genome sequence of a human enterovirus 71 strain isolated in Brunei in 2006 was determined. Phylogenetic analysis based on the complete genome sequence classified this strain into subgenogroup B5.

## GENOME ANNOUNCEMENT

Human enterovirus 71 (HEV71), a member of the human enterovirus A species of the family *Picornaviridae*, is an emerging pathogen that has recently become a serious health threat in the Asia-Pacific region. Although infection normally causes mild illness that is often undiagnosed, HEV71 has emerged as the dominant cause of large outbreaks of hand, foot, and mouth disease (HFMD) and can cause serious central nervous system infections leading to aseptic meningitis and encephalitis with a very high mortality.

A pattern of increased epidemic activity and endemic circulation of HEV71 has been observed in the region since 1997 and is associated with the regular emergence of new genetic lineages of HEV71. Genogroup A is represented by a single virus strain, the prototype HEV71 isolate *BrCr*, isolated in the United States in 1969 (1). The genogroup B lineages B1 and B2 circulated between 1970 and 1990 (2), and B3 through B5 have emerged in Southeast Asia since 1997 (3). A progenitor lineage, B0, has recently been proposed to describe isolates that circulated in Europe between 1963 and 1967 (4). Among the genogroup C lineages, C1 replaced B as the dominant genotype in Europe and America from 1987, C2 emerged in 1995, and C3 through C5 have been involved in epidemic activity in Southeast Asia since 1997 (5, 6). Genogroup D emerged as a single virus strain isolated in India in 2002 (7).

In 2006, Brunei reported its first major outbreak of HEV71 infection, which was associated with fatalities from neurologic complications. More than 1,681 children were affected, with 3 deaths resulting from severe neurologic disease (8). In this study, we performed complete genome sequencing of the HEV71 strain BRU-2006-35334, isolated from a patient with uncomplicated HFMD in Brunei during the 2006 outbreak.

The clinical isolates were initially plaque purified and propagated in RD cells up to at least five passages for amplification and sequencing. Fifteen synthetic oligonucleotide primer pairs were designed to amplify overlapping fragments that span the whole genome of HEV71, based on the alignment of available full-length sequences of different HEV71 genogroups. Full-length sequence assembly was performed using Sequencher version 4.7 (Gene Codes Corporation). The BRU-2006-35334 genome was found to be 7,412 nucleotides (nt), excluding the poly(A) tail. The 5′ untranslated region (5′ UTR) was found to be 747 nt, followed by an open reading frame (ORF) consisting of 2,194 codons, encoding a polyprotein, and a 3′ UTR that was 83 nt long. Phylogenetic analysis of the complete genome sequence of HEV71 clustered BRU-2006-35334 into subgenogroup B5.

Brunei is surrounded entirely by the East Malaysian state of Sarawak. Sarawak has experienced HEV71 outbreaks every three years (1997, 2000, and 2003), caused by the B3, B4, and B5 subgenogroups, respectively (9). All B5 subgenogroup isolates reported seem to have diverged from an ancestral strain, 5511-SIN-00, isolated in Singapore as early as 2000 (9). Subsequently, subgenogroup B5 emerged in Japan (10) and Sarawak (9) before appearing in Peninsular Malaysia and Brunei in 2006.

### Nucleotide sequence accession number.

The full-length sequence of the HEV71 subgenogroup B5 isolated in Brunei in 2006 has been deposited in GenBank under accession number JN992282.

## References

[1] Schmidt NJ Lennette EH Ho HH 1974 An apparently new enterovirus isolated from patients with disease of the central nervous system. J. Infect. Dis. 129:304–309 4361245 10.1093/infdis/129.3.304

[2] McMinn PC 2002 An overview of the evolution of enterovirus 71 and its clinical and public health significance. FEMS Microbiol. Rev. 26:91–107 12007645 10.1111/j.1574-6976.2002.tb00601.x

[3] Huang SW Hsu YW Smith DJ Kiang D Tsai HP Lin KH Wang SM Liu CC Su IJ Wang JR 2009 Reemergence of enterovirus 71 in 2008 in Taiwan: dynamics of genetic and antigenic evolution from 1998 to 2008. J. Clin. Microbiol. 47:3653–3662 19776232 10.1128/JCM.00630-09 PMC2772620

[4] van der Sanden S Koopmans M Uslu G van der Avoort H 2009 Epidemiology of enterovirus 71 in the Netherlands, 1963 to 2008. J. Clin. Microbiol. 47:2826–2833 19625480 10.1128/JCM.00507-09 PMC2738086

[5] Cardosa MJ Perera D Brown BA Cheon D Chan HM Chan KP Cho H McMinn P 2003 Molecular epidemiology of human enterovirus 71 strains and recent outbreaks in the Asia-Pacific region: comparative analysis of the VP1 and VP4 genes. Emerg. Infect. Dis. 9:461–468 12702227 10.3201/eid0904.020395 PMC2957976

[6] Solomon T Lewthwaite P Perera D Cardosa MJ McMinn P Ooi MH 2010 Virology, epidemiology, pathogenesis, and control of enterovirus 71. Lancet Infect. Dis. 10:778–790 20961813 10.1016/S1473-3099(10)70194-8

[7] Deshpande JM Nadkarni SS Francis PP 2003 Enterovirus 71 isolated from a cse of acute flaccid paralysis in India represents a new genotype. Curr. Sci. 84:1350–1353

[8] AbuBakar S Sam IC Yusof J Lim MK Misbah S MatRahim N Hooi PS 2009 Enterovirus 71 outbreak, Brunei. Emerg. Infect. Dis. 15:79–82 19116058 10.3201/eid1501.080264 PMC2660687

[9] Ooi MH Wong SC Podin Y Akin W del Sel S Mohan A Chieng CH Perera D Clear D Wong D Blake E Cardosa J Solomon T 2007 Human enterovirus 71 disease in Sarawak, Malaysia: a prospective clinical, virological, and molecular epidemiological study. Clin. Infect. Dis. 44:646–656 17278054 10.1086/511073

[10] Mizuta K Abiko C Murata T Matsuzaki Y Itagaki T Sanjoh K Sakamoto M Hongo S Murayama S Hayasaka K 2005 Frequent importation of enterovirus 71 from surrounding countries into the local community of Yamagata, Japan, between 1998 and 2003. J. Clin. Microbiol. 43:6171–6175 16333123 10.1128/JCM.43.12.6171-6175.2005 PMC1317214

